# Correction: The relationship between N-terminal pro-brain natriuretic and coronary artery lesions in Kawasaki disease: A meta-analysis

**DOI:** 10.1371/journal.pone.0354951

**Published:** 2026-07-28

**Authors:** Yanyan Li, Chaolong Zheng, Sisi Cheng, Limin Chu, Zhiqing Chen, Guiling Liu

The Funding statement for this article is incorrect. The correct Funding statement is as follows: This study was supported by the Medical Science Research Project of Hebei Provincial Health Commission (No. 20231035).
