# Supplementary material for: The relationship between N-terminal pro-brain natriuretic and coronary artery lesions in Kawasaki disease: A meta-analysis
Source: PLoS One. 2025 Jun 10;20(6):e0324552. doi: 10.1371/journal.pone.0324552 (PMC12151394; doi:10.1371/journal.pone.0324552)
Supplement: Attachment — Submitted filename: Response to Reviewers1_20250326121023.pdf [file pone.0324552.s003.pdf]

1、Although you mentioned that NT proBNP has higher diagnostic value in older children, can't we assume that younger children have narrower confidence intervals, which means higher test accuracy?The reference value for NT proBNP in infants under 1 year old is significantly higher, and this age group should be considered separately. Especially, infants under 6 months old have very high levels of NT proBNP. It is a fact that the incidence rate of coronary artery disease (CAL) in this period is high, but it does not directly increase with NT proBNP level. Please analyze and consider this point.

Answer： Kawasaki disease, cutaneous mucinous lymph node syndrome, coronary artery injury, infants, midbrain natriuretic peptide precursors were used as keywords to search CNKI, Wanfang and Wipu data to find related literature. Kawasaki disease, infantile, coronary artery injury, N-terminal natriuretic peptide precursors were searched in Pubmed and embase databases, but no related literature was found. The median age of all children in the literature included in this article was over 1 year, so it is particularly unfortunate that the study could not be conducted at 1 year or 6 months of age.

Subgroup analysis with age (< 3 years old and ≥3 years old), as covariates showed that the diagnostic value of NT-ProBNP combined with CAL was greater in the group older than 3 years old (6.4VS3.6). This may be because NT-proBNP levels are highest in the first few weeks

after birth and gradually decrease with age . It has also been reported that the level of NT-proBNP in infants (2442±1866 pg/mL) is significantly higher than that in children (945±1151 pg/mL) , so a high level of serum NT-proBNP concentration when children are over 3 years old is more valuable for predicting coronary artery injury in Kawasaki disease.
