# Supplementary material for: The relationship between N-terminal pro-brain natriuretic and coronary artery lesions in Kawasaki disease: A meta-analysis
Source: PLoS One. 2025 Jun 10;20(6):e0324552. doi: 10.1371/journal.pone.0324552 (PMC12151394; doi:10.1371/journal.pone.0324552)
Supplement: Attachment — Submitted filename: Response to Reviewers1.docx [file pone.0324552.s002.docx]

1、If the child's level is>1000, what recommendations does your meta-analysis have for clinical doctors? Should BNP routine examination be performed on all children with Kawasaki disease?

Answer：when NT-proBNP levels exceed 1,000 pg/mL in pediatric patients with Kawasaki disease, heightened vigilance for potential coronary artery damage and enhanced follow-up measures are warranted. An NT-ProBNP level ≥1000ng/L effectively predicts coronary artery damage.

2、Although coronary angiography and echocardiography are considered the gold standards for diagnosing coronary heart disease, these methods have inherent limitations and lack sufficient sensitivity.

Answer： Although echocardiography and coronary angiography are the gold standard for diagnosing coronary artery injury, many young children are difficult to soothe because they need complete quiet cooperation for echocardiography. Coronary angiography diagnosis requires interventional operation, which has great side effects and high cost for children, and many families of children have concerns.

3. Due to the nature of the research design, a Chinese specific literature database was used. However, is it possible to include reference materials that cannot be accessed in English? If the references are only provided in Chinese, the reviewers will not be able to evaluate their content.

Answer：Marked after reference (Chinese)

4. In this study, although the evaluation of whether sufficient NT proBNP information was provided in patient selection seems sufficient, the review of the definition of KD, the definition of CAL, and the methods for evaluating CAL is insufficient. This is one of the main focuses of the research

Answer：Kawasaki disease (KD), also known as cutaneous mucosal lymph node syndrome, is a systemic non-specific vasculitis that occurs in infants and young children, mainly involving small and medium-sized blood vessels. The main clinical symptoms are fever, mucositis, and rash. The incidence rate of Kawasaki disease (KD) in Southeast Asia has been increasing year by year for more than 20 years. Early onset of Kawasaki disease is often characterized by fever, which may be accompanied by clinical manifestations such as cracked lips, hard swelling at the ends of hands and feet, and rash. Coronary artery disease (CAL)is the most serious complication.of Kawasaki disease, including coronary artery dilation, aneurysms, cardiomyopathy, etc, and is currently the main cause of secondary acquired heart disease in children.In particular, coronary artery disease is often detected 2-3 weeks after onset，Many children have developed coronary artery lesions at the time of diagnosis, posing great challenges to treatment. Moreover, the severity of CAL lesions and the intervention difficulty in the sequelae stage of Kawasaki disease are even higher than that of adult coronary heart disease,resulting in the decline of the quality of life of children, and bring serious family economic burden. Although echocardiography and coronary angiography are the gold standard for diagnosing coronary artery injury, many young children are difficult to soothe because they need complete quiet cooperation for echocardiography. Coronary angiography diagnosis requires interventional operation, which has great side effects and high cost for children, and many families of children have concerns . At present, there is still a lack of early simple and accurate assessment of CAL in children with Kawasaki disease. Therefore, it is of great clinical significance to find indicators for early diagnosis of KD and prevention of CAL. In recent years, some studies have confirmed that increased plasma NT-proBNP levels in the acute phase of KD is a high risk factor for coronary disease , and a significant positive correlation with coronary artery diameter, providing a basis for early diagnosis of coronary artery lesions in Kawasaki disease, but some studies suggest that N-terminal brain sodium peptide precursor (NT-ProBNP) is meaningless or controversial for predicting coronary artery lesions.Although the existing systematic evaluation study NT-ProBNP evaluation kawasaki disease coronary artery lesions are valuable, but its included literature before 2020, and less literature, after 2020 recently published relevant literature, and the incidence of kawasaki disease in recent years, to update its diagnostic value, provide evidence-based basis for early clinical diagnosis of kawasaki disease and prevention of coronary artery lesions.

5、Although you mentioned that NT proBNP has higher diagnostic value in older children, can't we assume that younger children have narrower confidence intervals, which means higher test accuracy?The reference value for NT proBNP in infants under 1 year old is significantly higher, and this age group should be considered separately. Especially, infants under 6 months old have very high levels of NT proBNP. It is a fact that the incidence rate of coronary artery disease (CAL) in this period is high, but it does not directly increase with NT proBNP level. Please analyze and consider this point.

Answer：Due to the inclusion of patients with a median age of 1 year or older in the literature, it is not possible to use 1 year as a cutoff for calculation.

Subgroup analysis with age (< 3 years old and ≥3 years old), country (China and Japan), and detection method (chemiluminescence and ELISA) as covariates showed that the diagnostic value of NT-ProBNP combined with CAL was greater in the group older than 3 years old (6.4VS3.6). This may be because NT-proBNP levels are highest in the first few weeks after birth and gradually decrease with age [41,42]. It has also been reported that the level of NT-proBNP in infants (2442±1866 pg/mL) is significantly higher than that in children (945±1151 pg/mL) [43], so a high level of serum NT-proBNP concentration when children are over 3 years old is more valuable for predicting coronary artery injury in Kawasaki disease.
